# Preliminary Toxicological Analysis in a Safe-by-Design and Adverse Outcome Pathway-Driven Approach on Different Silver Nanoparticles: Assessment of Acute Responses in A549 Cells

**DOI:** 10.3390/toxics11020195

**Published:** 2023-02-20

**Authors:** Giulia Motta, Maurizio Gualtieri, Melissa Saibene, Rossella Bengalli, Andrea Brigliadori, Marie Carrière, Paride Mantecca

**Affiliations:** 1 Department of Biotechnology and Biosciences, University of Milano-Bicocca, Piazza della Scienza 2, 20126 Milan, Italy; 2 Research Centre POLARIS, Department of Earth and Environmental Sciences, University of Milano-Bicocca, 20126 Milan, Italy; 3 Department of Earth and Environmental Sciences, University of Milano-Bicocca, Piazza della Scienza 1, 20126 Milan, Italy; 4 National Research Council of Italy, Institute of Science, Technology and Sustainability for Ceramics (CNR-ISSMC former CNR-ISTEC), Via Granarolo 64, 48018 Faenza, Italy; 5 Univ. Grenoble-Alpes, CEA, CNRS, IRIG, SyMMES, CIBEST, 38000 Grenoble, France

**Keywords:** nano-enabled products, adverse outcomes pathway, safe-by-design, in vitro lung cells, nanotoxicity, silver nanoparticle hazard

## Abstract

Silver nanoparticles (Ag NPs) are among the most widely used metal-based nanomaterials (NMs) and their applications in different products, also as antibacterial additives, are increasing. In the present manuscript, according to an adverse outcome pathway (AOP) approach, we tested two safe-by-design (SbD) newly developed Ag NPs coated with hydroxyethyl cellulose (HEC), namely AgHEC powder and AgHEC solution. These novel Ag NPs were compared to two reference Ag NPs (naked and coated with polyvinylpyrrolidone—PVP). Cell viability, inflammatory response, reactive oxygen species, oxidative DNA damage, cell cycle, and cell–particle interactions were analyzed in the alveolar in vitro model, A549 cells. The results show a different toxicity pattern of the novel Ag NPs compared to reference NPs and that between the two novel NPs, the AgHEC solution is the one with the lower toxicity and to be further developed within the SbD framework.

## 1. Introduction

Silver nanoparticles (Ag NPs) are among the most widely used metal-based nanomaterials (NMs) for several applications (e.g., for food packaging, cosmetics, textiles, and health care). Such important use is mainly due to their antimicrobial properties [1]. In fact, thanks to their antibacterial capability [1], Ag NPs are nowadays used in several fields, from the textile industry to biomedical application [2,3]. Moreover, their use as antimicrobial materials is gaining relevance for their capability to combat pathogens causing infections in vitro and in vivo [4].

Ag NPs are present in different products, health care and fitness, cleaning, food packaging, household equipment, electronic devices and even toys [5,6]. These widespread uses inevitably increase the possibility of accidental release of these NPs to the environment, with a consequent increase in the exposure of humans and other organisms [7]. The various routes of exposure to Ag NPs for humans are therefore multiple: ingestion, inhalation, dermal contact and, at times, directly in systemic circulation via intravenous injection.

Although the detailed anti-pathogenic mechanism of Ag NPs remains to be fully clarified, nano-enabled products (NEPs) based on Ag NPs are of interest for their capability to exert antimicrobial functions through microbial membrane and microbial subcellular structure damages (i.e., mitochondria, ribosomes, and vacuoles), caused by the release of free Ag^+^ ions and consequent formation of reactive oxygen species (ROS) [8]. The ROS production is therefore a key feature of the antibacterial properties of Ag NPs, but this may pose a hazard to human and other organisms, if cell damage occurs in unwanted species. When considering human health, several mechanisms have been proposed to explain how Ag NPs exert their toxicity. NMs may cause inflammatory response or reactive oxygen species (ROS) production; these are processes that can alter the cell membrane and damage organelles [9]. It has been observed [10] that Ag NPs induce ROS production and cell apoptosis through a caspase-dependent intracellular pathway in liver hepatocellular adenocarcinoma cell line (HepG2). Other authors also observed the induction of ROS production and a reduction in glutathione (GH) after Ag NP exposure due to the release of free Ag^+^ ions. The increase in ROS caused adverse effects on cell viability and cell membrane integrity in several cell lines, both human and murine [11]. Therapeutic synthesized Ag NP exposure showed a dose- and time-dependent inhibition of cell viability, cell proliferation and cell morphology in A549 cells because of the increased oxidative stress [12]. The increase in ROS and subsequent cell death in Ag NP-exposed cells was also related to the formation of autophagosomes and autolysosomes and to a decrease in mitochondrial transmembrane potential (MTP) [13]. 

Noteworthy, after entering in contact with cells, NPs can undergo different possible transformations in terms of their pristine physico-chemical (p-chem) properties; for several metal oxide NPs, dissolution has been reported as a major process, and agglomeration and other surface modifications are reported to play a key role in NP effects [14]. 

Although these are possible drawbacks, NPs and nano-enabled products (NEPs, i.e., new products in which NPs are intentionally added to improve specific properties of the product or to substitute materials of fossil origin) are gaining relevance in everyday life. In light of this, to reduce the uncertainty of the potential adverse impact of NPs or NEPs on human health and the environment, already starting from the first steps of nanomaterial (NM) conceptualization and production and onwards, the application of the safe-by-design (SbD) strategy has been proposed and applied [15]. As reported by the authors, among the different toxicological tests suggested for a SbD approach, viability (by MTT, XTT, MTS and WST or Alamar Blue or neutral red) and the generation of reactive oxygen species (such as using 2′-7′-dichloro-fluorescin—DCFH) should be considered. In addition to this, the authors suggest considering additional biological endpoints, such as inflammation, and the stability of the NM itself. In this context, and in view of the 3Rs principle, in vitro studies are gaining prominent relevance to collect significant data to sustain the lack or reduce the unwanted and undesired intrinsic hazards of NPs and NEPs, in a life cycle-oriented approach. 

In this study, four different Ag NPs were selected to investigate how their p-chem properties might modulate the interactions with cells in a simple in vitro system (human alveolar adenocarcinoma A549 cells in monoculture). The particles selected have a similar nominal diameter but different surface coating agents. Two Ag NPs, namely the NPs coated with hydroxyethyl cellulose (HEC), were developed ex novo while two other NPs are commercially available, namely the naked Ag (used here as reference Ag NPs) and the NPs coated with polyvinylpyrrolidone (PVP). The novel AgHEC were synthetized in solution (AgHECs) and dried as a powder (AgHECp). The novel NPs were thoroughly characterized with different analytical methods to provide their relevant chemical and physical properties; all the Ag NPs were characterized, prior the toxicological exposures, in terms of size, shape, surface charge and agglomeration state to provide a common characterization, useful for understanding the biological effects. The acute toxic effects and the influence of Ag NP properties on A549 responses (cell viability, cell death, inflammatory response, ROS production and bio-interactions between cells and Ag NPs) after 24 h of exposure were evaluated. The biological endpoints were selected according to the adverse outcome pathway (AOP) 173 (https://aopwiki.org/aops/173 accessed on 9 January 2023) being a molecular-initiating event (MIE) or a key event (KE) determining the final adverse outcome (AO) of the AOP, that is lung fibrosis. Indeed, this AOP was recently reported to appropriately describe the toxicological impact of some NMs that interact with cell membrane components (e.g., receptors and lipids) (MIE) and lead to lung fibrosis, such as carbon nanotubes or cerium oxide nanoparticles [16,17,18,19]. This AOP would also possibly describe the toxicological pathways of Ag NPs, which have been reported to induce lung fibrosis [20]. In addition to these endpoints, DNA damage (assessed by γH2AX) and cell cycle alteration were considered as outcomes of interest for further assessing the possible adverse effects of Ag NPs on lung epithelial cells, and because DNA damage is a consequence of oxidative stress and inflammation while cell cycle arrest is a consequence of DNA damage.

## 2. Materials and Methods

### 2.1. Chemicals and Reagents

All chemicals and reagents were purchased from Sigma Aldrich (Milano, Italy) if not stated elsewhere. In the framework of the ASINA European project, we selected different Ag NPs. Ag naked (#484059, AgNKD) and Ag with PVP surface coating (#576832, AgPVP) were purchased from Sigma Aldrich (Milano, Italy) as benchmark materials for toxicological profiles comparison against the ASINA produced NMs, namely Ag with HEC doping obtained in suspension or in powder form (hereinafter defined as AgHECs and AgHECp, respectively). These two NPs were kindly provided by the Italian National Research Council (ISSMC-CNR, former ISTEC-CNR, Faenza, Italy). Briefly, a solution of AgNO_3_ 0.05 M (Sigma-Aldrich, Milan, Italy) was mixed and stirred for five minutes with a solution of hydroxyethyl cellulose (Dow Chemical, Midland, MI, USA) to a final molar ratio Ag/HEC of 5.5. The hydrogel was formed by adding a 1 M solution of NaOH (Sigma-Aldrich, Milan, Italy). The final nanosol dispersion was obtained after 24 h from hydrogel formation by adding MilliQ water. The Ag HEC nanosol was in case granulated, by means of spray freeze drying, dehydrated to also obtain the AgHEC powder sample. More details on AgHEC NP preparation are reported in [21,22,23,24,25]. Reference NPs AgNKD and AgPVP were obtained according to [25].

### 2.2. NP Suspension Preparation

Ag NP suspensions for characterization and treatments were prepared in MilliQ water to reach an initial stock suspension of 1 mg/mL of Ag NPs (considering the same mass of Ag content for all the tested NPs). For AgNKD and AgPVP NPs in powder form, the following sonication method was followed: NP suspensions, prepared in sterile glass vials or a 50 mL falcon tube, were put in an insulation box filled with ice and sonicated by means of an ultra-sonicator (Sonopuls HD3100, Bandelin, Berlin, Germany) equipped with a 2 mm probe. NP suspensions were sonicated by applying in total 40 W for 10 min (1 s pulse, 1 s pause cycle). AgHECs, after vortexing the stock suspension for 30–60 s with an angle of 45°, were directly diluted in MilliQ water to reach the desired concentration. AgHECp, after being weighed, were pre-wet NPs with a few mL (0.5–1 mL) of ultrapure MilliQ water, vortex for 30–60 s with an angle of 45° and left to set for at least 30 min (better overnight), then the desired amount of MilliQ water was added to reach the concentration of 1 mg/mL. No sonication was applied for these two NPs. All NP stock suspensions were characterized for their stability over time and kept at 4 °C. For NP characterization in MilliQ water or in cell culture medium, stock suspensions (up to 1 moth old) were vortexed and diluted to obtain the desired concentrations.

### 2.3. Ag NP Characterization

The novel Ag NPs, namely AgHECs and AgHECp, were submitted prior to their use in toxicological experiments to a set of analytical characterizations. Morphology, crystalline structure, and particle size were characterized by transmission electron (TEM) analyses using a FEI (Hillsboro, OR, USA) Tecnai F20 microscope operating at 200 keV. AgHECp was dispersed in isopropyl alcohol and sonicated for 15 min. AgHECs is sonicated for 15 min. The obtained suspensions are deposited on a perforated carbon film supported by a gold grid. The preparation was then dried at 40 °C. Phase contrast images were recorded to evaluate the morphology of the nanoparticles. High resolution (HR-TEM) and selected area electron diffraction (SAED) were used to study the crystalline domains. The electron microscope was also equipped with the STEM accessory, these pictures were recorded using a high-angle annular dark-field (HAADF) detector and then they were used for the size distribution analysis.

X-rays diffraction (XRD) was performed with a with Bruker (Billerica, MA, USA) D8 Advance (Cu Kα 1.5406 Å), working conditions: 2θ interval 10–80°, step 0.04°, step time 0.5 s. Few droplets of AgHECs suspension (500 mg/L) were deposited on a glass substrate and dried at 80 °C, the procedure was repeated to obtain a homogenous layer. AgHECp was directly pressed into the sample holder.

UV–Vis absorption properties of the AgHEC NPs were recorded by a Perkin Elmer (Waltham, MA, USA) Lambda 750 spectrophotometer. AgHECs was diluted to 6 mg/L with MilliQ while AgHECp was dispersed in MilliQ at the concentration of 6 mg/L. The solutions were placed in a quartz cuvette and directly submitted to analysis. 

Finally, Fourier transform infrared spectroscopy (FTIR) spectra were acquired by a Thermo Scientific (Waltham, MA, USA) Nicolet iS5 equipped with iD7 an attenuated total reflectance (ATR, with a diamond window) by directly using the AgHECs and AgHECp in their pristine forms, i.e., as particles solution (5000 mg/L) or as powder. The following parameters were considered during the FTIR analysis: scan range 4000–420 cm^−1^, resolution 0.121 cm^−1^ and twenty-four scans per sample acquisition.

Ag NP suspensions for toxicological analyses were characterized in terms of size, shape, surface charges (ζ-potential), agglomeration state and dispersion by Dynamic Light Scattering (DLS) analysis using the Zetasizer Nano ZS90 (Malvern Ltd., Warwickshire, UK) and by transmission electron Microscopy (TEM) by a Jeol JEM (Jeol Ltd., Tokio, Japan). Ag NPs were prepared in MilliQ water or cell culture medium (DMEM supplemented with 10% *v*/*v* of fetal bovine serum, FBS) also considering two different working temperatures, RT for samples in MilliQ and 37 °C for sample in DMEM. For DLS analysis, Ag NP suspensions were prepared at the concentration of 10 and 100 µg/mL. All the suspensions were analyzed at time 0, just after preparation, and after 24 h of incubation at RT to assess NP stability in solution. Regarding TEM analysis, Ag NP suspensions were prepared in MilliQ water at the concentration of 100 µg/mL; 5 µL of suspension were deposited on a TEM grid (Formvar-carbon support film, 200 mesh, copper) and let dry overnight. All the samples were observed under the Jeol Jem 2100 Plus TEM Microscope (Jeol Ltd., Tokio, Japan). 

### 2.4. Cell Culture

Human alveolar epithelial cells (A549 cell line, ATCC^®^ CCL-185, American Type Culture Collection, Manassas, VA, USA) were cultivated (passages between 9 and 25) in DMEM medium (Sigma Aldrich, Milano, Italy) supplemented with 10% fetal bovine serum (FBS; Gibco Life Technologies, Monza, Italy) and antibiotics (penicillin/streptomycin, 100 U/mL; Euroclone, Pero, Italy). Cells were maintained in an incubator at 37 °C and 5% CO_2_. Cells were treated with different concentrations of Ag NPs (0.1–1–10–20–50–100 µg/mL) in submerged condition for 24 h and then processed for further analysis. Untreated cells were considered as negative control. Routinely mycoplasma detection was performed as reported in Appendix A  and Figure A7.

### 2.5. Viability Assay

The viability of the cells was assessed through the Alamar Blue assay (Invitrogen Life Technologies, Monza, Italy) and MTT assay (described in Appendix A) (although its limitation with Ag NPs reported in [26]. Cells were seeded on a 6 multiwell plate (2.5 × 10^5^ cell/well); after 24 h, cells were treated with different concentrations of Ag NPs (0.1–1–10–20–50–100 µg/mL, in DMEM medium with 1% serum content) for 24 and 48 h and untreated cells were considered as Negative Control. After the exposure to NPs, cell medium was removed, and cells were washed with PBS. 800 µL of cell culture medium with 10% of the Alamar Blue test solution were added to each well. Cells were then incubated at 37 °C and 5% CO_2_ for 2 h to allow the viable cells to reduce resazurin in resorufin. Then, 200 µL from each well were pipetted in triplicate in a 96-well black plate and the fluorescence was measured at an excitation wavelength of 560 nm and a gain of 82 with a TECAN Infinite M200 Pro microplate reader (TECAN, Männedorf, Switzerland). The emission at 590 nm was recorded and the viability expressed as relative variation over the control ratio. To evaluate specific cell death pathways, namely necrosis and apoptosis, A549 cells were seeded on 6 multiwell plate (2.5 × 10^5^ cell/well) and treated for 24 h with different concentrations of Ag NPs (10–20–50 µg/mL); untreated cells were considered as negative control. At the end of the exposure, cells were rinsed with phosphate buffered saline (PBS), detached by gently trypsinization and stained with Annexin V and Propidium Iodide (PI). Cytofluorimetric analysis (CytoFLEX, Beckman Coulter, Cassina de Pecchi, Italy) was then performed on cell pellets by analyzing the green (FITC channel) and red fluorescence (ECD channel) of 10.000 cells per sample. 

### 2.6. Inflammatory Response

Cells were seeded on 6 multiwell plates at the density of 2.5 × 10^5^ cell/well and after 24 h they were treated with different concentrations of Ag NPs (0.1–1–10–20–50–100 µg/mL). The release of Interleukin 8 (IL-8) was evaluated in the supernatants collected after 24 h of exposure, centrifuged at 1200 rpm for 6 min and then stored at −80 °C until analysis. The quantification of released IL-8 was performed with IL-8 ELISA matched antibody pair kit (Invitrogen, Life Technologies, Monza, Italy) according to the manufacturer’s instruction. The sample absorbance was measured by a multiplate reader (Infinite 200 Pro, TECAN, Männedorf, Switzerland) at the wavelength of 450 nm; the concentration of interleukins was calculated based on standard curves and data were shown as *p* g/mL. Untreated cells were considered as negative control. 

### 2.7. Intracellular ROS

The intracellular ROS level was measured using 2′,7′-dichlorodihydrofluorescein diacetate (H_2_DCFDA, Thermo Fisher Invitrogen, Waltham, MA, USA) probe. A549 cells were seeded (2.5 × 10^5^ cell/well) in a 6 multi-well cell culture plate and incubated overnight. Cells were treated with 20 and 50 µg/mL of Ag NPs for 90 min and 24 h. We exposed two wells for each concentration to evaluate the background fluorescence in absence of the probe. H_2_O_2_ 0.03% was used as positive control. When the treatment was removed, cells were washed with PBS. Depending on the well, they were loaded with PBS alone or containing 10 µM of probe for 20 min in the dark at 37 °C. When the solution was removed, cells were washed with PBS twice, detached using trypsin and collected by centrifugation (1200 rpm, 6 min). Fluorescence was measured immediately with a CytoFlex (Beckman Coulter, Cassina de Pecchi Italy) using an excitation wavelength of 488 nm and an emission wavelength of 525 nm and measuring 10,000 events for each sample. The fluorescence intensity of cells not treated with the probe was subtracted to the respective treated cells to have the real fluorescence emission.

### 2.8. DNA Damage

γH2AX was evaluated as a marker for DNA double-strand breaks (DDS). The phosphorylation of the histone H2AX is in fact related to the formation of DDS in response to several toxicant, oxidative stress and after cell cycle arrest [27,28] and γH2AX has been proposed as the most informative marker of double-strand breaks [29]. A549 cells were seeded (2.5 × 10^5^ cells/well) in a 6-well cell culture plate and incubated overnight. Cells were treated with 20 or 50 µg/mL of silver NPs for 24 h or with etoposide (1.65 µM) as a positive control. At the end of the treatment, cells were washed with PBS, collected by centrifugation, fixed using 4% PFA for 15 min and permeabilized with ice-cold 90/10% methanol/PBS for 10 min. The samples were stained using Phospho-Histone H2A.X (Ser139) (20E3) Rabbit mAb (Alexa Fluor^®^ 488 Conjugate) (Cell Signaling Technology, Danvers, MA, USA) following the manufacturer instructions. γH2AX fluorescence intensity was measured using flow cytometry (CytoFlex, Beckman Coulter, Cassina de Pecchi, Italy). Fluorescence was measured immediately using an excitation wavelength of 488 nm and an emission wavelength of 525 nm and measuring 10,000 events for each sample.

### 2.9. The Cell Cycle

Cell cycle analysis was performed by staining the DNA with PI followed by flow cytometry. A549 cells were seeded (2.5 × 10^5^ cells/well) in a 6-well cell culture plate and incubated overnight. Cells were treated with 20 and 50 µg/mL of Ag NPs for 24 h. Etoposide (1.65 µM) was used as a positive control. Then, the suspension was removed, cells were washed with PBS, collected by centrifugation, and suspended in ice-cold ethanol/PBS solution (90%/10% *v*/*v*). The cells were suspended in PBS containing 20 µg/mL of RNase DNase-free inhibitor (Sigma-Aldrich, Milan, Italy) for 30 min at 37 °C. PI 10 µM was added and the samples were analyzed using a CytoFlex (Beckman Coulter, Cassina de Pecchi, Italy) with an excitation wavelength of 488 nm and an emission wavelength of 610 nm and measuring 10,000 events per sample.

### 2.10. Cell–Particle Bio-Interaction

Quantitative analysis: Cells were seeded on 6 multiwell plates at the density of 2.5 × 10^5^ cell/well and after 24 h, they were treated with different concentrations of Ag NPs (1–10–20–50 µg/mL) for additional 24 h. At the end of the exposure, the cells were recovered by trypsinization, and the samples were analyzed using a CytoFlex (Beckman Coulter, Cassina de Pecchi, Italy) with an excitation wavelength of 488 nm. The side scatter signal (SSC) of the gated cell population was recorded as proxy variable of the particle–cell interaction (both at cell membrane and/or after internalization). 

Qualitative analysis: A549 cells were seeded on coverslip in 6 well plates (3 × 10^5^ cell/well). After 24 h, cells were exposed with 20 µg/mL of AgHECp, and AgPVP for an additional 24 h. At the end of the exposure, cells were washed 2 times with sterile PBS and fixed in a solution of 2.5% glutaraldehyde in phosphate buffer (pH 7.4) for 1 h. Then, after washings in the same buffer, cells were post-fixed in 1% osmium tetroxide aqueous solution for 2 h at 4 °C in the dark. Dehydration in ethanol (50, 70, 90, 96 and 100%) and infiltration in epoxy resin were the following steps. Embedded samples were cut with an ultramicrotome (Reichert-Jung Ultracut E) to obtain thin sections (60–70 nm) to be observed under the transmission electron microscope (TEM). Before observation, the sections were stained for 30 min with an aqueous solution of Uranyl Acetate (1%). 

### 2.11. Statistical Analysis

Data are expressed as the mean ± standard error (SE) of at least three biological independent experiments (N > 3, if not otherwise stated). Fold change values were log2 transformed and reported and analyzed as such. Statistical analyses were performed using R software [30], using one-way ANOVA test followed by Dunnett’s post hoc test if the homogeneity of variance was confirmed by Levene’s test; conversely, the pairwise Wilcox test was applied to determine statistical differences; values of *p* < 0.05 were considered statistically significant.

## 3. Results

### 3.1. Novel AgHEC Particles Characterization

**Figure 1:**
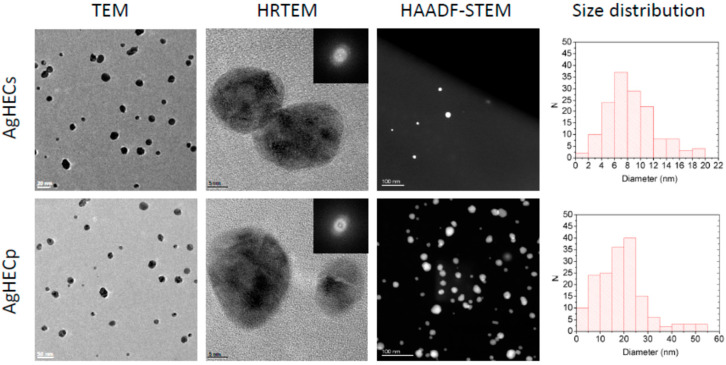
Transmission electron microscopy images of AgHEC NPs. TEM pictures were used for the morphological characterization of the NPs, HRTEM were used to determine the diffraction patterns of the novel particles while the HAADF-STEM to determine the size distribution of the particles.

The novel AgHEC particles were specifically designed, and a series of different analytical approach were used to characterize their morphological and functional properties. TEM analysis allowed to characterize the morphology and the crystalline structure of the NPs (Figure 1). Both AgHECs and AgHECp have a rather narrow size distribution (3–20 nm and 5–50 nm for AgHECs and AgHECp, respectively) and their diffraction patterns (HRTEM-SAED analysis) confirmed their crystalline structure, formed by twinned domain’s structure.

**Figure 2:**
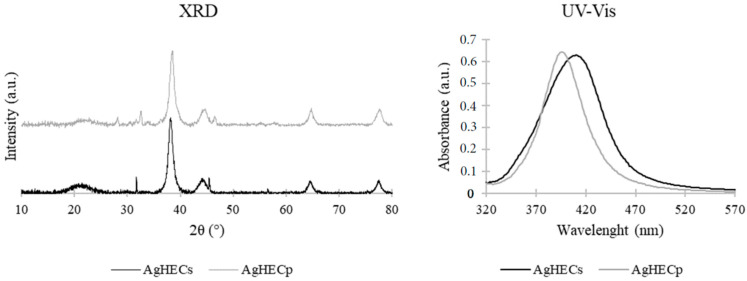
X-ray diffraction and UV–Vis spectra of novel NPs. XRD peaks typical of Ag NPs are reported together with other minor peaks related to the synthesis process. The UV–Vis absorption spectra agree with the silver core nature of the novel NPs.

The XRD results (Figure 2) show a typical XRD pattern of Ag NPs, the main peaks detected can be indexed as a Face-Centered Cubic (FCC) structure (JCPDS, file no. 4-0783). The patterns obtained show the presence of diffraction peaks at 38, 44, 64 and 77°, corresponding, respectively, to (111), (200), (220) and (311) Ag planes.

Crystallite size was determined using the Scherrer method on the main diffraction peak (111). AgHECs has a crystallite size of 8.8 nm, and AgHECp of 9.1 nm. The two samples show similar crystallite size and the AgHECs value is aligned with the TEM size (9 nm), while TEM analysis for AgHECp showed a larger size (19 nm). This may be due to the spray freeze-drying leading to an aggregation, but the low temperature does not allow the recombination of crystal seeds. 

Other diffraction peaks may be related to synthesis byproducts, mainly sodium chloride (NaCl), while the amorphous region at approximately 20°, more evident for AgHECs, is due to the amorphous glass substrate. UV–Vis absorption spectra showed the typical Surface Plasmon Resonance peak of Ag NPs. In this case, the maximum absorption falls at 410 nm for AgHECs and at 397 nm for AgHECp, typical wavelengths of spheroidal Ag NPs with a size of approximately 10–20 nm.

**Figure A1:**
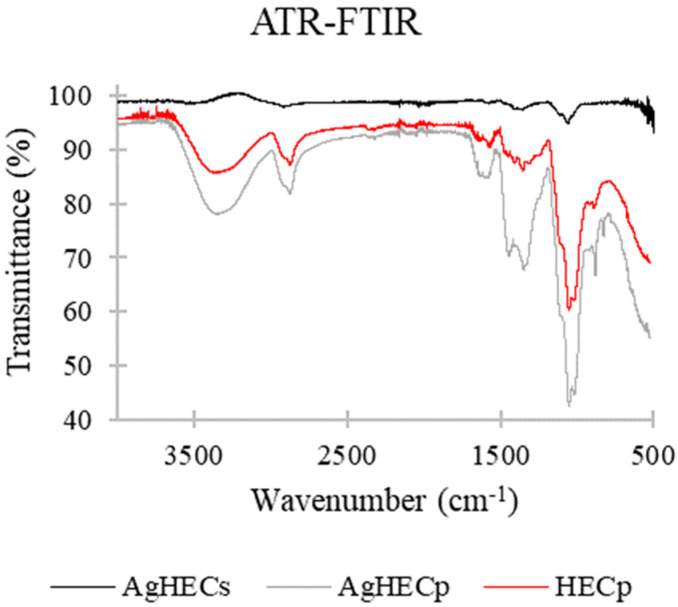
FTIR spectra of the AgHEC NPs. The ATR–FTIR spectrum of the hydroxyethyl cellulose (HEC) functionalized with quaternary ammonium used as capping agent (in red) show a broad band at approximately 3500–3200 cm^−1^, which refers to stretching of hydroxyl group. Peaks at 2870 and 3010 cm^−1^ refer to C–H stretching. The band at approximately 1650–1580 cm^−1^ is related to the amine N–H bending. Peaks at 1440 and 1350 cm^−1^ refer to C–H and O–H bending. The alcohol C–O stretching peaks at 1110 and 1050 cm^−1^ are observed. Using this one as reference, it is possible to observe that AgHECp follows well the relative intensity of the absorption peaks. The AgHECs spectrum is influenced (very low signal intensity) by the water suspension.

FTIR spectra confirmed the presence of the HEC shell around the NPs. The AgHECs gave lower signals due the presence of the dispersant (water). Cleared peaks were recorded for the AgHECp (Appendix B, Figure A1)

### 3.2. Ag NP Characterization for Toxicological Analyses

Ag NPs physical properties in the different solutions tested were comparable considering the two concentrations (10 and 100 µg/mL, Table 1). Ag NP hydrodynamic diameters showed a general tendency to reduce over the time in both MilliQ and DMEM solutions; among the different NPs, the AgHECs and AgHECp NPs showed a lower tendency to modify their hydrodynamic diameter over 24 h, when dispersed in aqueous solution compared to the reference NPs (AgNKD) (Table 1). 

The ζ-potential resulted highly negative for AgNKD (−27.57 mV), slightly negative for Ag-PVP and Ag-HECs (−6.07 mV and −4.71 mV, respectively) and positive for AgHECp (+9.92 mV). This differential surface property of the different NPs is related to the different coating—the naked particle being the most negative compared to the coated ones—and to the coating procedure, the ζ-potential of HECs and HECp being different. 

**Figure 3:**
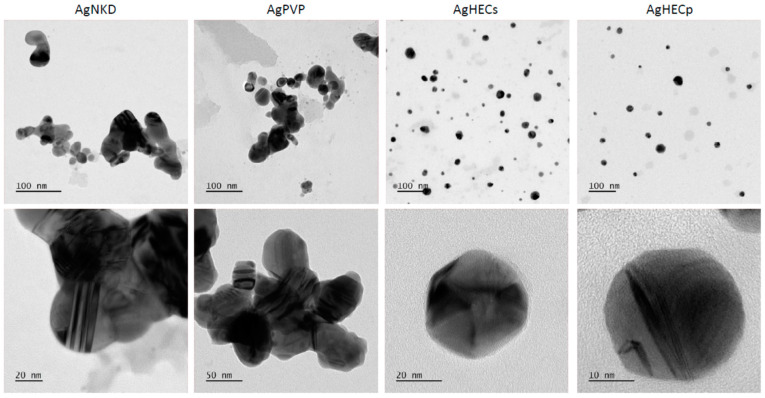
Transmission electron microscopy images of Ag NP suspensions. Ag NPs in MilliQ water were analyzed by TEM. The images in the upper panel show how particle suspensions are dispersed and in the lower panel is shown a detail of small agglomerates or single particles.

Ag NP suspensions, prepared in MilliQ water, were also analyzed by transmission electron microscopy (TEM) to qualitatively evaluate their morphology and agglomeration state (Figure 3). All the Ag NPs showed primary particles with a spherical shape in the range of 20 to 30 nm. AgHECs and AgHECp resulted better dispersed compared to AgNKD and AgPVP, which were characterized by agglomerates in the order of hundreds of nm, as already observed by DLS analysis. This difference in agglomeration is a relevant outcome of the different surface modifications which allow for a better dispersion during manufacturing processes, such as spray coating of textile, at the same time, agglomeration state greatly affects bio-interactions and effects in living cells.

### 3.3. Cell Viability

**Figure 4:**
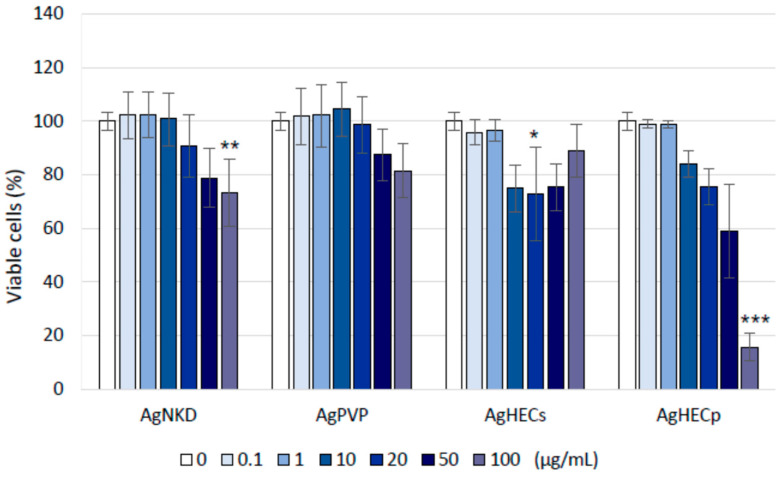
Cell viability. The graphs show the percentages of cell viability compared to the negative control, assessed by Alamar Blue assay after 24 h of treatment. Data are presented as the mean of at least three independent experiments ± SE. Statistical analysis: one-way ANOVA followed by Dunnett’s test. * *p* < 0.05 compared to control; ** *p* < 0.01 compared to control; *** *p* < 0.001 compared to control group.

**Figure A2:**
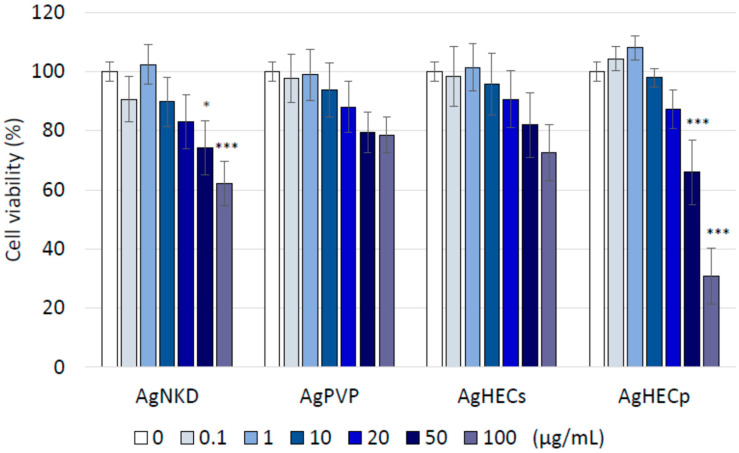
Cell viability assessed by MTT. Significant reduction of cell viability was observed after AgHECp and AgNKD. AgPVP and AgHECs particles were effective less. ANOVA one way followed by Dunnett test, * *p* < 0.05; *** *p* < 0.001 compared to control cells, N = 3.

**Figure A3:**
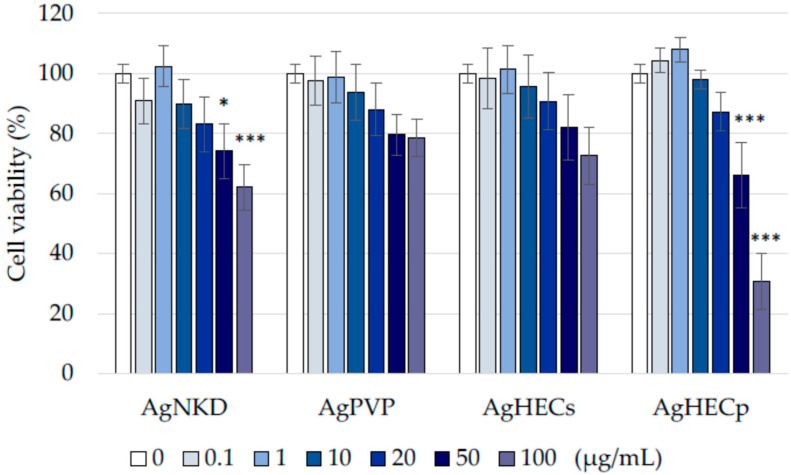
Cell viability assessed by Alamar Blue at 48 h. A significant reduction in cell viability was observed after AgHECp and AgNKD. AgPVP and AgHECs particles were less effective. One-way ANOVA followed by Dunnett’s test; * *p* < 0.05; *** *p* < 0.001 compared to control cells. N = 3.

To better assess cell viability, avoiding possible NP interference, Alamar Blue and MTT assays were tested. Finally, the Alamar Blue method was selected as the best performing assay (Figure 4, MTT data in Appendix B, Figure A2). Viability decreased concentration-dependently for all the particles tested, but the AgHECs, with a clearer effect for the AgHECp NPs. Similar results were obtained after 48 h of treatment, showing no additional toxicity to exposed cells (Appendix B, Figure A3). Given the general low cytotoxicity of the Ag NPs, the 24 h IC_50_ was correctly calculated only for AgHECp (IC_50_ equal to 57.05 µg/mL, with an upper and lower confidence values of 47.61 and 70.01 µg/mL) while for the other Ag NPs we assume that the IC_50_ is higher than 100 µg/mL. 

**Figure A4:**
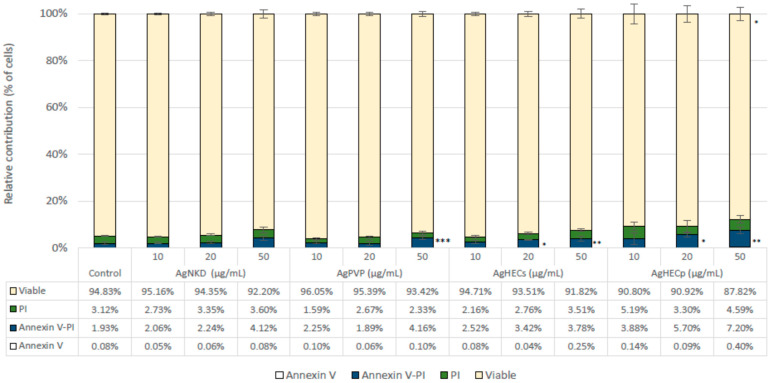
Cell death characterization after exposure to Ag NPs. The percentages of viable cells, apoptotic cells (Annexin V positive), late apoptotic cells (Annexin V/PI—positive) and necrotic cells (PI—positive). A549 cells after exposure for 24 h to 50 µg/mL to Ag NPs; each treatment is reported with its relative negative control (unexposed cells). Statistical analysis: one-way ANOVA followed by *t*-test; * *p* < 0.05; ** *p* < 0.01, *** *p* < 0.001 compared to control cells. N = 3.

According to its higher cytotoxic effects, AgHECp (Appendix B, Figure A4) also induced a concentration-dependent increase in annexin V/PI positive necrotic/late apoptotic cells that was statistically significant at 20 and 50 µg/mL (5.7% and 7.2% versus 1.9% in control cells) with a consequent reduction in viable cells (90.92% and 87.8% com-pared to 94.8% in control cells). Additionally, AgHECs and AgPVP induced a statistical increase in necrotic/late apoptotic cells (3.4 and 3.7% at 20 and 50 µg/mL for AgHECs and 4.1% at 50 µg/mL for AgPVP) while the AgNKD showed no significant effect at the concentrations we tested (Appendix B, Figure A4). 

### 3.4. Reactive Oxygen Species Formation

**Figure 5:**
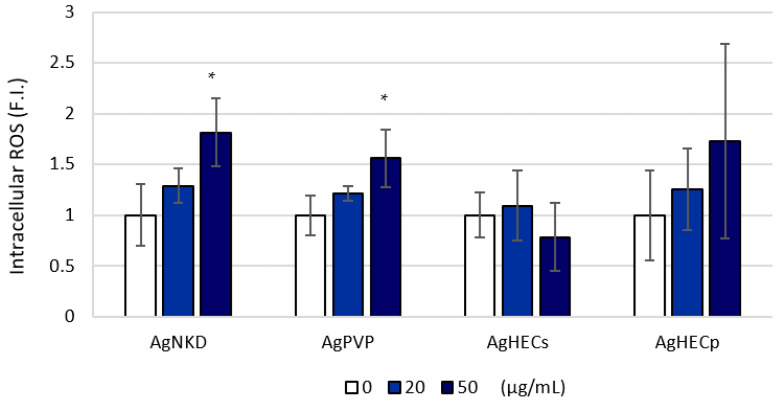
Reactive oxygen species were significantly increased after 90 min of treatment in AgNKD and AgPVP exposed cells (50 µg/mL). Data are presented as the mean of at least three independent experiments ± standard deviation. Statistical analysis: one-way ANOVA followed by Dunnett’s test. * *p* < 0.05 compared to control non exposed cells.

**Figure A5:**
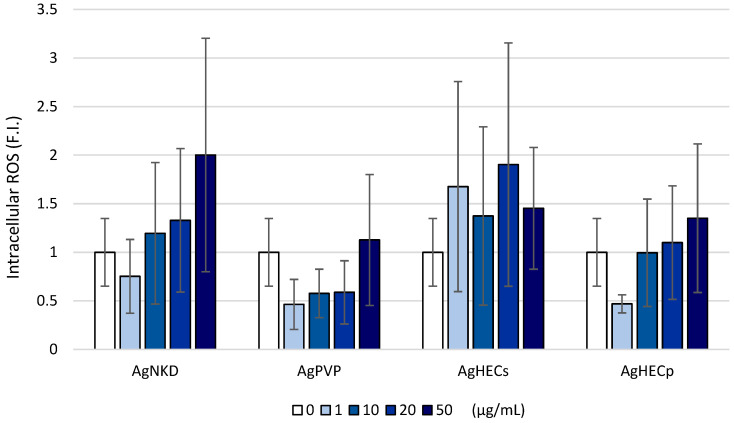
ROS levels in cells exposed to Ag NPs for 24 h. ROS expression was assessed using H_2_DCFDA as indicator although some modulation statistically significant effects were not observed. Data are presented as the mean ± SE. Statistical analysis: one-way ANOVA followed by *t*-test.

Intracellular ROS formation was selected to investigate the capability of the NPs to increase the oxidative status in exposed A549 cells. H_2_DCFDA conversion to fluorescent DCFA was assessed by cytofluorimetric assay. After 90 min of treatment, AgNKD and AgPVP at the exposure concentration of 50 µg/mL induced a significant increase in intracellular ROS. A non-significant increase was observed in AgHECp exposed cells, while absence of modulation was observed in AgHECs treatments (Figure 5). After 24 h of exposure to Ag NPs, ROS were slightly but not statistical significantly modulated by all the NPs (Appendix B, Figure A5).

### 3.5. Inflammatory Response (IL-8 Release)

**Figure 6:**
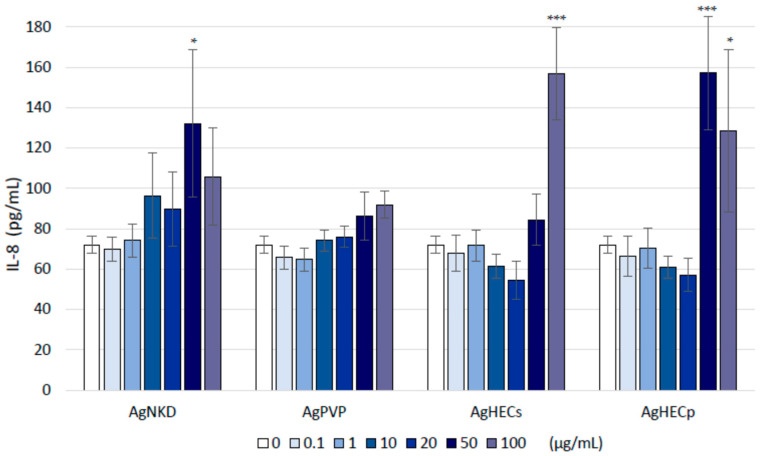
The inflammatory response (IL-8 release). IL-8 protein concentration was differently modulated by the NPs. Higher releases were observed at the higher exposure concentration of the AgHEC NPs (ANOVA followed by Dunnett’s test) and at the concentration for 50 µg/mL AgHECp. Data are presented as the mean of the pg/mL release by each sample (n = 3) ± SE of at least three independent experiments. * *p* < 0.05 and *** *p* < 0.001 compared to control group.

The release of the inflammatory mediator (IL-8) in A549 cells after 24 h of exposure to Ag NPs was modulated differently by the different NPs (Figure 6). Although some increase in the pg/mL of IL-8 in treated samples, statistically significant increases were observed only for the higher concentrations of AgHECs (100 µg/mL) and at the concentration for 50 µg/mL of AgHECp. Among the different NPs, AgPVP was the least active in inducing IL-8 modulation. After 48 h of exposure, the modulation of the inflammatory protein IL-8 was not significant at the concentrations tested (data not shown).

### 3.6. Oxidative DNA Damage (γH2AX)

**Figure 7:**
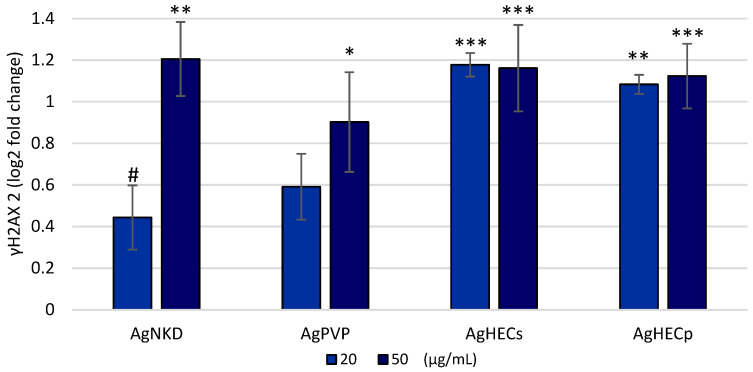
DNA damage (expressed as log2 FC of γH2AX) was determined by quantifying the increase in the γH2AX protein. Control values are equivalent to the zero line, values above this value are actual increases in the protein content while negative values are downregulation of the protein. Increases in DNA damages in exposed cells were observed for all the Ag NPs at the concentration of 20 and 50 µg/mL. Data are presented as the mean of at least three independent experiments ± SE. Statistical difference analyzed by one-way ANOVA and Dunnett’s test. * *p* < 0.05 compared to control; ** *p* < 0.01 compared to control, ANOVA with post hoc; *** *p* < 0.001 compared to control, ANOVA with post hoc; # *p* < 0.05 compared to the 50 µg/mL exposure concentration.

The DNA damaging effects of the Ag NPs was assessed by the quantification of the phosphorylated protein H2AX (γH2AX). The increase in the fluorescent signal of γH2AX (Figure 7) after A549 treatment with the different NPs was significant for all the NPs at the concentrations tested (20 and 50 µg/mL). Compared with the other NPs, the AgHEC NPs showed a high increase in H2AX phosphorylation after the exposure to 20 µg/mL.

### 3.7. Cell Cycle Alteration

**Figure 8:**
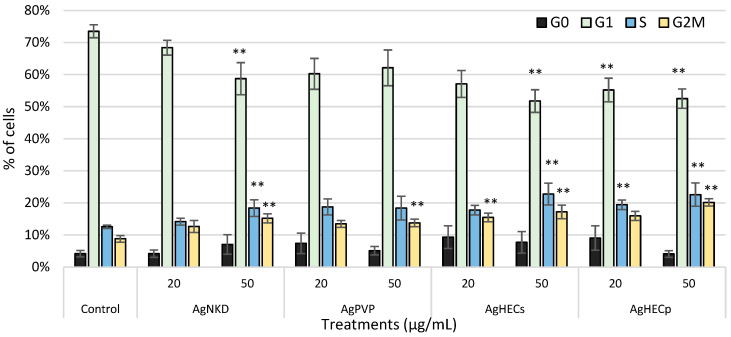
Cell cycle analysis. In the graph are reported the percentages of cells in the different cell cycle phases: subG0, G1, S and G2M (n > 3). It is possible to observe a statistically significant increase in cells in G2M phase after treatment with all the Ag NPs. Data are presented as the mean of at least three independent experiments ± SE. Statistical analysis: one-way ANOVA followed by Dunnett’s test; ** *p*-value = 0.01 compared to control.

Since damage at the DNA is a relevant endpoint in assessing the hazards of new NPs, we also tested the capability of the different NPs to induce alteration in the cell cycle progression (Figure 8). The cell cycle may be altered by different events, an increase in cells stalled in the S phase is normally related to issues with the DNA replication machinery or the presence of DNA damages that slow the replication process, while increases in cells in the G2/M phase may be still related to the presence of DNA damages to be corrected prior to mitosis or to alteration to the mitosis machinery. The results show that after treatment with the different Ag NPs at the concentration of 50 µg/mL a significant increase in cells blocked in G2/M was observed and coupled to an increase in S phase (except for the AgPVP NPs). Again, the AgHECp NPs were able to induce cell cycle alterations at a lower concentration. 

### 3.8. Cell–Particle Bio-Interactions

**Figure 9:**
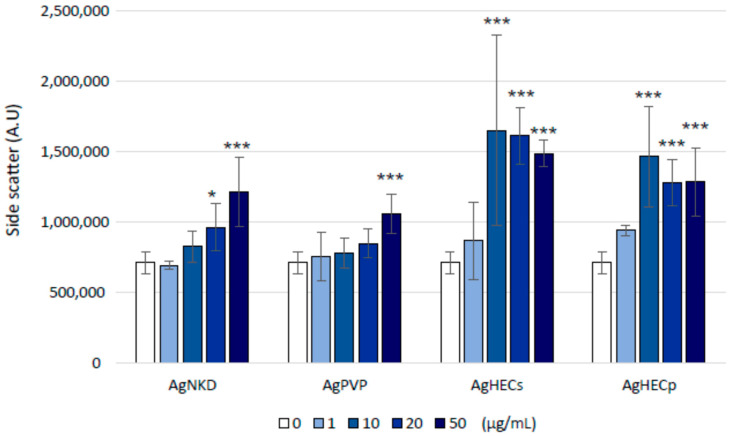
Bio-interaction analysis. The side scatter (SSC) values of cells exposed at different concentrations of the different Ag NPs. Clear differences among the NPs are reported, with AgHEC NPs showing higher SSC values compared to AgNKD and AgPVP treatments. Data are presented as the mean of at least three independent experiments ± SE. Statistical analysis: one-way ANOVA followed by Dunnett’s test; * *p*-value = 0.05; *** *p*-value = 0.001 compared to control.

**Figure A6:**
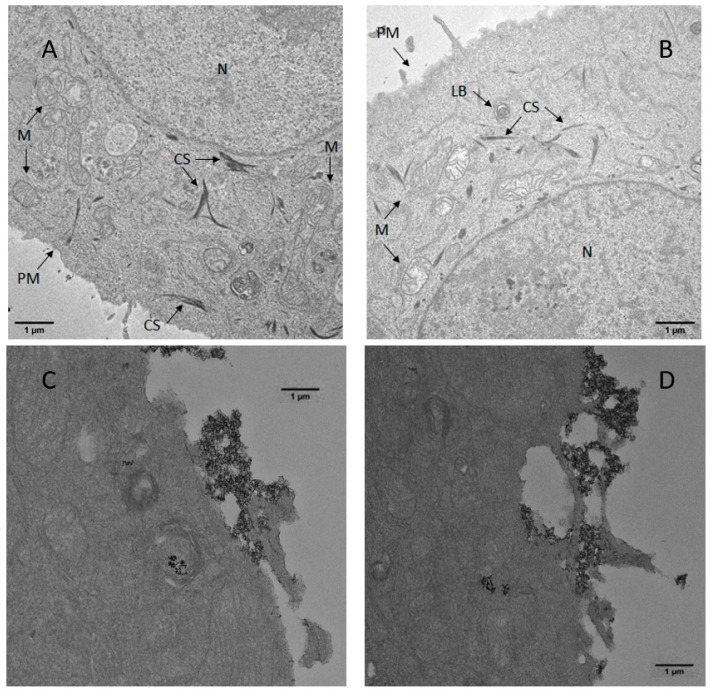
TEM picture of A549 exposed to AgPVP (**B**) or AgHECp (**C**,**D**) NPs. Increased membrane localization of NPs is evident in AgHECp treated cells and intracellular localization in lamellar bodies (**C**) in compared to AgPVP (**B**) and control cells (**A**). M = mitochondria, PM = plasma membrane, LB = lamellar bodies, CS = cytoskeleton, and N = nucleus.

**Figure A7:**
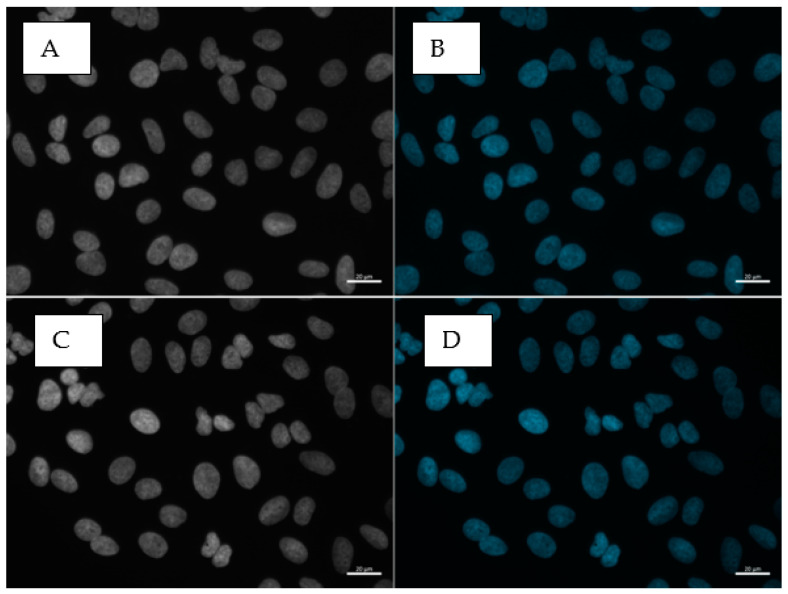
Hoechst stained A549 for mycoplasma screening. The absence of extranuclear cyto-plasmic staining confirm the absence of mycoplasma in the cultured A549 cells. Pictures are from two different passages: passage 10 is reported in Figure A6 (**A**) (tones of grey channel) and (**B**) (Hoechst channel), passage 19 is reported in Figure A6 (**C**) (tones of grey channel) and (**D**) (Hoechst channel).

To analyze the possible NP interactions and/or uptake of Ag NPs, monolayers of A549 treated for 24 h were investigated by laser beam scattering and TEM imaging. The SSC reported show (Figure 9) that A549 cells treated with AgNKD and AgPVP had the lowest scattering values and were statistically different from the control only at the higher concentration of exposure. On the contrary, cells treated with AgHECs and AgHECp resulted in higher SSC values and therefore higher cell–particle interactions (significant values different from the controls already at the concentration of 10 µg/mL (*p* < 0.001). TEM imaging (Appendix B, Figure A6) supports these findings. AgPVP treated cells show an ultrastructure well maintained, and different subcellular structures are recognizable. AgHECp cells on the contrary clearly show particles internalization and particle membrane interactions. In the future, a more detailed investigation of the intracellular localization of the NPs and possible modifications induced at ultrastructural levels may support the AOP-oriented investigation strategy.

## 4. Discussion

Safety of nanomaterials and nano-enabled products (NEPs) is a primary objective for more sustainable and innovative goods given the intrinsic potential hazards of objects in the nanometric scale [31,32]. The evaluation of the hazards of NPs of interest is usually performed after a synthesis procedure is set up to shape the NP of interest for the specific application it is intended for. Therefore, the toxicological evaluation has been usually performed at the end of the production, application, and disposal cycle to confirm that the particle or the product containing the particle does not pose a hazard to humans or the environment. To overcome this a posteriori evaluation of the risk for the human health and the environment of NPs or NEPs, safe-by-design (SbD) approaches have been proposed during the last years [15,33]. The basic concept is to provide a priori evidence of the absence of hazards in the new NP or NEP during their life cycle therefore minimizing or removing unwanted or unpredicted risk after exposure. One of the key questions of SbD procedure is to what extent they are predictive of potential chronic effects, given the absence of acute toxicity. To address this question, methodological approaches have been proposed such as in [33] to provide a standardized procedure to follow for different NMs with similar intended application. On the other hand, the definition of the adverse outcome pathways (AOPs) concept [34,35,36] provided a novel framework to support SbD approaches [15,16,37]. Within the framework of AOPs we identified AOP #173 related to lung fibrosis of substances interacting with the membrane components (e.g., receptors and lipids) of lung cells leading to fibrosis. In fact, the induction of lung fibrosis due to NP exposure has been proposed by several authors [16,17,18,19]. In our experiments, we synthetized novel Ag NPs coated with a shell of HEC. The thorough characterization performed, confirmed the crystalline nature of the novel NPs and the presence of the external layer of HEC. These novel particles were analyzed in parallel for their toxicological properties according the AOP selected. This AOP has, as a molecular-initiating event (MIE), the interaction of the substance with the cell membrane. The data reported confirm the interaction of Ag NPs with the plasma membrane of exposed cells and the internalization of clusters of NPs into membrane structures within the cytoplasm, interestingly this was more evident for AgHECp. Internalization of NPs is a relevant mechanism also for cytotoxicity. Indeed, the capability of some metal or metal oxide NPs, especially Ag NPs, to exert their adverse effects through the intracellular release of metal ions after internalization is largely reported [38,39,40,41]. Internalized NPs might interact with cellular macromolecules according to their affinity or by generating ROS [41,42]. The results here reported show a significant increase in ROS after 90 min of exposure. According to the expected oxidative burst usually reported few hours after exposure of in vitro or in vivo models to NPs [42,43,44,45]. We would like to speculate, that the absence of positive results with the HEC coated NPs, may be related to a quenching effect or a masking effect of the HEC over the fluorescent probe used to test ROS. In fact, according to the short life of reactive species in cells, we observed, also in HEC coated Ag NPs, the increase in γH2AX, a marker of oxidative damage of the DNA, that is in fact also related to precedent ROS formation [46,47]. ROS formation may also be related to inflammatory mediator release [48,49,50,51] and therefore contribute to the modulation of IL-8 we reported here. The activation of the inflammatory response in exposed cells is another key event (KE) in the AOP for lung fibrosis since chronic inflammation [52,53,54] is a trigger of changes in the extra cellular matrix leading to fibrosis [55]. We also report here the capability of Ag NPs to alter the cell cycle of the exposed epithelial cells, adding an additional event on the path to lung fibrosis [56]. What are relevant in the SbD concept are the differences we have observed between the differentially coated NPs (Appendix B, Table A1). Considering AgNKD as a reference we report here that the AgPVP nanoparticles are likely the less active in inducing adverse events related to the selected AOP. Interestingly, we report a clear difference of Ag NPs coated with hydroxyethyl cellulose (HEC), the focus of our study, considering two different conditions. The AgHECp was the most toxic, determining the highest cytotoxicity and IL-8 modulation, while the DNA damaging potency and the cell cycle alteration were similar between the two AgHEC NPs. The higher toxicity of AgHEC NPs could be related to both their higher stability and lower agglomeration in toxicological media, lower DLS hydrodynamic diameter and TEM images as compared to reference Ag NPs [56,57,58] and the direct effect of the HEC coating in favoring the internalization of the Ag NPs [57]. In the SbD framework and considering the AOP outcomes we reported here, the HEC coating being the core of the newly developed Ag NPs, the production and use of HEC coated Ag NPs should follow a wet production and use procedure rather than drying the NPs for subsequent uses. The reason why AgHEC in powder form is more hazardous than the original colloidal solution may be linked to the possible physico-chemical transformations of the coating polymer during the processes. Further, considering the methodology (freeze-drying) used to obtain AgHECp, very slight modifications of the polymer structure are expected. How such possible minor modifications may influence the biological reactivity is a fascinating aspect for future investigations.

## 5. Conclusions

**Figure 10:**
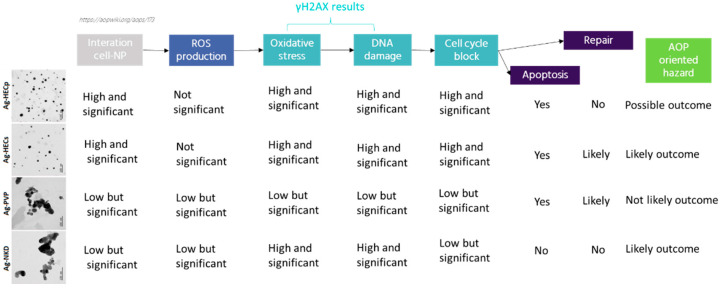
Summary of the AOP-oriented test analysis for the different NPs analyzed. Among the different NPs, the newly produced are the most likely to activate relevant events related to AOP #173, lung fibrosis, and therefore pose a relevant hazard for human health. The reference materials (AgNKD and AgPVP) are less relevant in the framework of the AOP selected. Significantly, the AOP events investigated clearly showed that the AgHECp are activating all the relevant events that may link the exposure to these NPs to the adverse lung outcome, while the AgHECs are less likely to determine such detrimental effects.

In conclusion, novel NPs (AgHEC) were here synthetized, characterized and tested in parallel for their hazards according to a SbD approach combined with relevant AOP events (Figure 9). The combination of these two relevant frameworks showed efficacy in characterizing the hazards of different Ag NPs and defining which production and use procedure should be considered with lower expected risk. The doping of the surface of Ag NPs seems to have a primary role in driving the toxicity of the newly synthetized particles and the selection of coated NPs with lower intrinsic hazards should be favored for subsequent testing and use in manufacturing procedure (Figure 10). Here, we show that HEC coating favors the dispersion of the Ag NPs in water-based media. This, from a manufacturing point of view, is a benefit since well-dispersed NPs are less prone to cloth and obstruct orifices needed for example during spraying. On the other hand, HEC coating favors particle–cell membrane interaction and cytotoxicity. Our results allowed the discrimination of the potential hazard of two different processes of AgHEC production and use, the wet (AgHECs) and dry (AgHECp) approaches, considering the first the one to favor for subsequent additional tests and manufacturing processes.

## Figures and Tables

**Table 1:** Ag NP characterization for cell exposure. Dynamic Light Scattering (DLS) analysis performed for Ag NPs (AgNKD, AgPVP, AgHECs and AgHECp) in MilliQ water and cell culture medium (CCM). The measurements were performed at two different time points, 0 and 24 h, and two concentrations (10 and 100 µg/mL) were considered. In the table are also reported the values of z-average (nm) ± SD and PDI ± SD. In addition, for each particle is indicated the value ζ-potential (mV) in MilliQ water at the concentration of 100 µg/mL.

NPs	Medium	Time (h)	µg/mL	z-Average (nm) ± SD	PdI ± SD
AgNKD ζ-potential: −27.57 (100µg/mL in mQ)	mQ	0	10	266.29 ± 35.96	0.47 ± 0.01
		24	10	142.52 ± 51.02	0.29 ± 0.05
		0	100	270.76 ± 53.18	0.45 ± 0.04
		24	100	109.35 ± 22.42	0.34 ± 0.08
	DMEM 1% FBS	0	10	624.32 ± 106.24	0.75 ± 0.09
		24	10	128.99 ± 15.32	0.24 ± 0.06
		0	100	328.71 ± 76.9	0.37 ± 0.13
		24	100	167.57 ± 7.55	0.33 ± 0.1
AgPVP ζ-potential: −6.07 (100µg/mL in mQ)	mQ	0	10	1515.88 ± 928.18	0.92 ± 0.14
		24	10	591.67 ± 192.93	0.81 ± 0.16
		0	100	695.91 ± 617.49	0.7 ± 0.26
		24	100	227.06 ± 159.62	0.45 ± 0.15
	DMEM 1% FBS	0	10	545.96 ± 386.17	0.69 ± 0.26
		24	10	165.92 ± 58.32	0.21 ± 0.15
		0	100	361.4 ± 110.43	0.43 ± 0.06
		24	100	185.11 ± 7.62	0.2 ± 0.14
AgHECs ζ-potential: −4.71 (100µg/mL in mQ)	mQ	0	10	122.04 ± 10.16	0.14 ± 0.02
		24	10	109.41 ± 8.36	0.15 ± 0.003
		0	100	122.16 ± 5.89	0.14 ± 0.02
		24	100	115.7 ± 5.37	0.15 ± 0.01
	DMEM 1% FBS	0	10	72.57 ± 6.26	0.23 ± 0.04
		24	10	77.82 ± 0.82	0.23 ± 0.03
		0	100	80.37 ± 2.4	0.2 ± 0.02
		24	100	75.23 ± 4.91	0.21 ± 0.05
AgHECp ζ-potential: 9.92 (100µg/mL in mQ)	mQ	0	10	293.83 ± 6.76	0.41 ± 0.06
		24	10	219.33 ± 5.13	0.4 ± 0.01
		0	100	304.89 ± 34.89	0.37 ± 0.07
		24	100	261.63 ± 26.86	0.36 ± 0.06
	DMEM 1% FBS	0	10	62.72 ± 10.85	0.46 ± 0.02
		24	10	148.66 ± 39.78	0.28 ± 0.03
		0	100	150.29 ± 19.7	0.35 ± 0.06
		24	100	88.63 ± 4.97	0.51 ± 0.01

## Data Availability

No data are at the moment available due to privacy requirements.

## Appendix A

- MTT assay

The viability of the cells was also assessed through the MTT assay. After the exposure to nanoparticles, cell medium was removed, and cells were washed with PBS. MTT was dissolved at 5 mg/mL in PBS, then a suspension containing 62.5 µL for each mL of cell culture medium (DMEM supplemented with 10% of FBS) was prepared and 1 mL of it was added to each well. Cells were then incubated in the dark at 37 °C and 5% CO_2_ for 2 h to allow the viable cells to reduce tetrazolium salt in formazan crystals. Then, supernatants were removed, and the precipitated crystals were solubilized using 1 mL of DMSO for each well. The plates were left for 10 min with continual shaking, then 200 µL from each well were pipetted in triplicate in a 96-well transparent plate and the absorbance was measured at 570 nm with a reference of 690 nm with a TECAN Infinite M200 Pro microplate reader. The viability is expressed as percent control ratio.

- Mycoplasma detection

The possible contamination by mycoplasma strains of the cultured cells is performed monthly according to [58]. Cells were seeded on a glass coverslip in 6 multiwell plate (2.5 × 10^5^ cell/well) and allowed to grow until reaching confluence. The medium is then removed, and cells are washed with sterile PBS. The cells are then fixed with 1 mL of paraformaldehyde fixative for 5 min, this procedure is repeated twice. Then, the fixative is removed, and the coverslips are allowed to dry under a sterile hood. After drying, cells are stained with Hoechst 33258 for 5 min. The stained cells are rinsed with sterile PBS and then are mounted with Prolong mounting solution. Each coverslip is analyzed under an AxioObserver.Z1 Cell imaging station (Zeiss, Jena, Germany), by an immersion oil 63x objective to detect the presence into the cytoplasm of small Hoechst positive extranuclear dots. At least 100 cells per coverslip are analyzed. Images are acquired by an AxioCam MRm digital camera and elaborated with the ZEN2.3 Blue software (Zeiss). Figure 6 reports representative pictures of A549 cells after Hoechst staining at two different passages of the cell culture, passage 10 and passage 19.

## Appendix B

**Table A1:** Summary of the toxicological outcomes and tentative ranking of the tested NPs from the less to the most toxic. Scores (in terms of number of +) were assigned considering the statistical significance of the effects, the dose response tendency of the effects and the first concentration of exposure giving a significant effect.

NPs	Cytotoxicity	ROS (90 min)	Inflammation	DNA Damage	Cell Cycle Alteration	Final Rank from Less to Most Toxic
AgNKD	**+**	**+**	**+**	**+**	**+**	**1**
AgPVP	**++**	**+**		**+**	**+**	**1**
AgHECs	**++**		**++**	**++**	**++**	**2**
AgHECp	**+++**		**++**	**++**	**++**	**3**
